# Eco-Friendly and COVID-19 Friendly? Decreasing the Carbon Footprint of the Operating Room in the COVID-19 Era

**DOI:** 10.3390/diseases11040157

**Published:** 2023-11-02

**Authors:** Christos Tsagkaris, Hamayle Saeed, Lily Laubscher, Anna Eleftheriades, Sofoklis Stavros, Eirini Drakaki, Anastasios Potiris, Dimitrios Panagiotopoulos, Dimos Sioutis, Periklis Panagopoulos, Ahsan Zil-E-Ali

**Affiliations:** 1 Public Health and Policy Working Group, Stg European Student Think Tank, Postjeskade 29, 1058 DE Amsterdam, The Netherlands; 2 Fatima Memorial Hospital College of Medicine & Dentistry, Lahore 54000, Pakistan; hamaylesaeed@gmail.com; 3 Department of Health Sciences, Swiss Federal Institute of Technology Zurich, 8092 Zurich, Switzerland; 4 Faculty of Medicine, National and Kapodistrian University of Athens, 115 27 Athens, Greece; 5 3rd Department of Ob/Gyn, Attikon University Hospital, National and Kapodistrian University of Athens, 124 62 Athens, Greeceapotiris@med.uoa.gr (A.P.);; 6 Department of Heart and Vascular Institute, The Pennsylvania State University College of Medicine, Hershey, PA 17033, USA

**Keywords:** climate change, environmental sustainability, pandemic preparedness, surgery, SARS-CoV-2

## Abstract

Surgery is one of the most energy-intensive branches of healthcare. Although the COVID-19 pandemic has reduced surgical volumes, infection control protocols have increased the ecological footprint of surgery owing to the extensive use of personal protective equipment, sanitation, testing and isolation resources. The burden of environmental diseases requiring surgical care, the international commitment towards environmental sustainability and the global efforts to return to the pre-pandemic surgical workflow call for action towards climate-friendly surgery. The authors have searched the peer-reviewed and gray literature for clinical studies, reports and guidelines related to the ecological footprint of surgical care and the available solutions and frameworks to reduce it. Numerous studies concede that surgery is associated with a high rate of energy utilization and waste generation that is comparable to major non-medical sources of pollution. Recommendations and research questions outlining environmentally sustainable models of surgical practices span from sanitation and air quality improvement systems to the allocation of non-recyclable consumables and energy-efficient surgical planning. The latter are particularly relevant to infection control protocols for COVID-19. Paving the way towards climate-friendly surgery is a worthy endeavor with a major potential to improve surgical practice and outcomes in the long term.

## 1. Introduction

Operating rooms are considered the most resource-intensive area of healthcare facilities. Their conduction requires up to 600% more energy than other hospital wards. This is due to the parallel functioning of electronic devices such as respirators, monitors and surgical tools, the accumulation of waste from medications, consumables, protective equipment and biohazardous material, the use of sterilization to decontaminate the infrastructure and the constant transportation of patients, healthcare professionals and material. The cumulative environmental pollution associated with these activities and their supporting functions such as documentation and archiving, supply chains, financial transactions and so forth, can be defined as the carbon footprint of surgical operations (CFSO) [1]. Studies that were conducted before the COVID-19 pandemic have provided estimations of the CFSO and recommendations on how to decrease them [1,2].

The emergence of the COVID-19 pandemic acted as a game-changer urging for reconsideration of the existing understanding of the CFSO. Globally, the surge of SARS-CoV-2 infections and hospitalizations led to a marked restriction of elective surgical services [3]. Although the absolute number of surgical operations performed has decreased, both the WHO and national or regional health bodies have endorsed an increase in intraoperative sanitation and personal protective equipment (PPE), in order to decrease the risk of infections and protect patients and healthcare professionals [4]. In addition, surgical teams have also been required to operate on COVID-19-positive patients or patients without a confirmed COVID-19 test result in a wide range of emergencies, particularly in acute trauma, obstetrics and gynecology [5]. Hence, although the volume of surgical operations has reduced during the pandemic, the ecological footprint of each operation has significantly increased.

Whether the cumulative ecological footprint of surgical care has increased during the pandemic remains debatable. However, the restriction of surgical care during COVID-19 in combination with the mounting effects of climate change on health tends to increase surgical morbidity. Simultaneously, the ecological footprint of surgical care spirals upward fueling a vicious circle of diseases requiring energy and waste-intensive surgical treatment. The continuous emergence of SARS-CoV-2 strains and novel pathogens (Monkeypox, hepatitis of unknown etiology) has made energy-intensive surgical resources (PPE, testing kits, etc.) a “new normal”.

On these grounds, it is high time to reconsider the environmental burden of surgery and the existing solutions for climate-friendly surgical practice. This paper provides a critical summary of the existing evidence and elaborates on its adaptation to the COVID-19 health crisis aiming to address the following matters of inquiry:

- Identify the ecological footprint of surgery and its contribution to healthcare waste;
- Review the impact of COVID-19 on the carbon footprint of operating rooms;
- Identify eco-friendly practices that can be implemented in operating rooms to decrease their carbon footprint;
- Evaluate the feasibility and effectiveness of these eco-friendly practices in the context of the COVID-19 pandemic;
- Provide recommendations for healthcare facilities to adopt eco-friendly practices in their operating rooms to reduce their carbon footprint.

## 2. Materials and Methods

To identify the relevant peer-reviewed publications and gray literature, the authors searched PubMed-Medline and Cochrane Library-Cochrane Central Register of Controlled Trials (CENTRAL) until 30 March 2023. The reference lists of the selected sources and relevant systematic reviews were also hand searched to identify potentially relevant resources. The search terms “surgery”, “carbon footprint”, “energy utilization”, “healthcare waste”, “ecological footprint”, “operating room”, “surgical treatment”, and “COVID-19” were used in combination with Boolean operators (AND, OR), when appropriate. Studies were included if they fulfilled all the following eligibility criteria: (1) ongoing or published studies reporting energy utilization, waste generation and overall ecological footprint of facilities and services related to surgical care, and (2) studies, reports and guidelines regarding the reduction of the ecological footprint of surgical care. A study was excluded if it met at least one of the following criteria: (1) non-English publication language, (2) study types: editorials, opinion articles, perspectives, and letters to the editor. No sample size restriction was applied when screening for eligible studies. Disputes in the selection of relevant studies were discussed between the two primary authors and a senior author until a consensus was reached. The literature was searched and reported according to the Preferred Reporting Items for Systematic Reviews and Meta-Analyses (PRISMA) extension for Scoping Reviews (PRISMASc) [6].

## 3. Results

To the best of our knowledge original studies reporting measures to decrease the carbon footprint of surgical operations conducted in accordance with COVID-19 safety protocols have not been published within the timeline of the search. Nevertheless, numerous studies have reported environmentally sustainable practices applicable to respiratory infection control in surgical settings.

### 3.1. The Carbon Footprint of Surgical Operations

Several single- or multicentric studies carried out by institutions based in the United Kingdom (UK), the United States (US), Canada and India have attempted to estimate the carbon footprint of surgical operations. The major contributors to the CFSO are greatly debated. Some studies focused on the intraoperative use of energy-intensive devices and consumables, while other estimations included the environmental burden arising from perioperative diagnostic processes, hospitalization, and postoperative care [1,2]. Electricity consumption has been widely acknowledged as the principal contributor to the CFSO [2]. Fewer studies indicated procurement and single-use items as the biggest source of environmental pollution [7].

**Figure 1:**
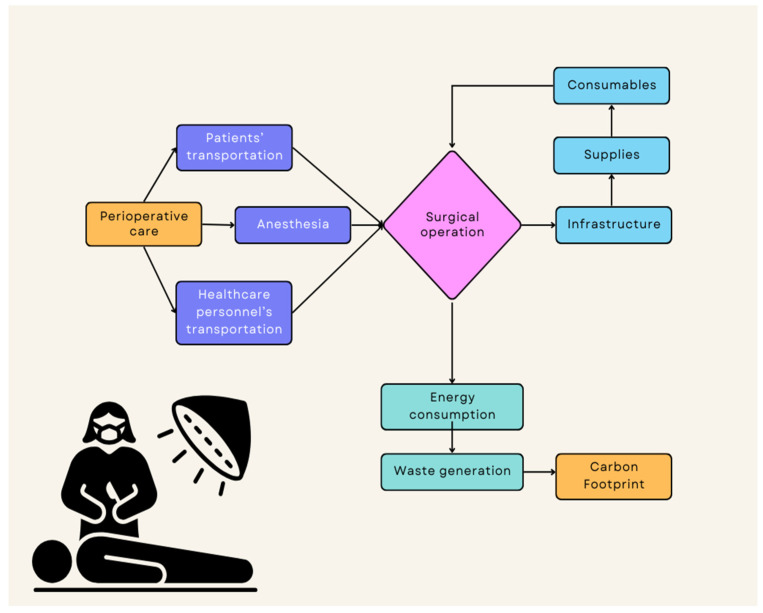
The carbon footprint of surgical operations.

The estimations of the CFSO ranged from 6–814 kg carbon dioxide (CO_2_) equivalents per operation—with ca 60 kg CO_2_ equivalents being the average ecological footprint of a commercial flight of one hour according to the publicly available calculator of the United Nations [1,7,8]. In general, robotic and laparoscopic surgery were more energy-consuming in comparison to open surgery. The same applied to lengthy procedures involving multiple organs. Relevant studies have shown significant differences in the carbon footprint of abdominal surgery compared to operations involving only one organ (breast implants, cataract eye surgery) [9]. Anesthetic gasses were also an important factor of environmental pollution with desflurane resulting in the environmental burden to multiply tenfold compared to sevoflurane or isoflurane [2], by reason of the shorter lifetime of gasses like sevoflurane in the atmosphere [2]. To the authors’ best knowledge, no study has evaluated the carbon footprint of processes supporting the function of the operating room. It was found that the procurement and transportation of patients and healthcare personnel accounted for more than 30% of the medical waste of acute medical care and more than 20% of ambulatory healthcare services, including surgical emergencies [10]. Major carbon hotspots in operating theatres are electricity use and procurement of consumables, single-use items, which cause two-thirds of the surgery carbon footprint, energy use in surgery, particularly in laparoscopic surgeries that employ energy-based devices and waste generation in operating theatres [2] (Figure 1).

In a carbon footprint analysis conducted for products used in the five highest volume surgical operations performed in the National Health System in England, the mean average carbon footprint of products was measured in CO_2_e (carbon dioxide equivalents). The average carbon footprint of products used for carpal tunnel decompression was 12.0 kg CO_2_e, 11.7 kg CO_2_e for inguinal hernia repair, 85.5 kg CO_2_e for knee arthroplasty, 20.3 kg CO_2_e for laparoscopic cholecystectomy, and 7.5 kg CO_2_e for tonsillectomy. To be sure, 23% of product categories were responsible for 80% of the carbon footprint of the five operations. The single-use hand drape (carpal tunnel decompression), single-use surgical gown (inguinal hernia repair), bone cement mix (knee arthroplasty), single-use clip applier (laparoscopic cholecystectomy), and single-use table drape (tonsillectomy) contributed the most carbon for each operation type. Production of single-use items contributed 54%, decontamination of reusables 20%, waste disposal of single-use items 8%, packaging production for single-use items 6%, and linen laundering 6%, on average [11].

Changes in practice and policy should be directed towards the products that contribute the most, such as reducing single-use items and switching to reusables, as well as optimizing processes for decontamination and waste disposal, which have been shown to reduce the carbon footprint of these operations by 23–42% [7].

In total, surgical operations have been held accountable for up to 70% of healthcare-related waste. To understand the magnitude of this number it is necessary to note that healthcare constitutes the second highest source of waste globally [7,12,13,14]. Based on these numbers and calculations, the current recommendations for the mitigation of healthcare-related environmental pollution include rationalizing energy consumption, prioritizing environmentally sustainable surgical supplies, improving the management of pharmaceutical waste [15], segregating operating rooms’ waste and reprocessing disposables [12].

### 3.2. Review of Previous Recommendations

In the pre-COVID-19 era, elaborate recommendations for the operating room environment were already in place. According to the Global Guidelines for the Prevention of Surgical Site Infection, published by the World Health Organization in 2018, the operating room ventilation system was designed to perform several functions: the primary one being to offer optimal thermal conditions for the patient and personnel and to maintain consistent air quality by removing aerosols and particles from the room. Secondary to ensure that specific air pressure requirements are met between connecting rooms. In the operating room, specialized ventilation units supplying filtered air at positive pressure are requisite. Approximately twenty air changes per hour are needed to disperse microbes created in the operation room and to prevent infiltration from adjacent regions. The most used ventilation system in well-resourced operating rooms is a turbulent flow of air. These systems homogenize fresh air with the operating room’s air, aerosols and particles. This causes faster dilution of the air volume and random particle movement. Laminar airflow systems with unidirectional fresh air flow at a constant velocity are widely utilized in environments where particle contamination is a significant problem, such as orthopedic implant surgery. In many countries, high-efficiency particulate air filters (at least 99.97% efficient in eliminating particles up to 0.3 μm in diameter) in operating rooms are required by law. According to local standard procedures, based on the manufacturer’s instructions and international guidelines, the operating room ventilation system should be checked, and filters changed on a regular basis [16].

The Centers for Disease Control (CDC) and the Healthcare Infection Control Practices Advisory Committee (HICPAC) also have joint comprehensive guidelines for environmental infection control in healthcare facilities which was updated in July 2019 and has a section dedicated to infection control and ventilation requirements for operating rooms. Pertinent guidelines include positive-pressure ventilation maintenance, ≥15 air changes per hour (of which ≥3 should be fresh air) and filtration of all fresh and recirculated air through designated filters with a minimum of 90% efficiency. In rooms devoid of laminar airflow engineering, air should be introduced at the ceiling and exhausted near the floor. Doors of the operating room should be kept closed except for necessary passage of equipment, personnel, and patients [17].

Of special interest are the guidelines in due consideration of TB patients, as these were, in the pre-COVID-19 time: usage of an N95 respirator without exhalation valves approved by the National Institute for Occupational Safety and Health and operating room doors to be closed until 99% airborne contaminants are eliminated after intubating a TB positive patient. In addition, placement of a bacterial filter between the anesthesia circuit and the patient’s airway should be ensured when anesthetizing a patient with confirmed or suspected tuberculosis, allowing enough time for air changes to clean 99% of airborne particulates from the air if the patient must be extubated in the operating room because extubating causes coughing. For infectious TB patients who require surgery, portable, industrial-grade HEPA (high-efficiency particulate air) filters should be used for momentary additional air cleaning during intubation and extubation. Positioning of the units should be such that all the air filling the room moves through the filter; place the unit in accordance with engineering recommendations. Lastly, scheduling operations of infectious TB patients as the day’s final surgical cases to maximize the time available for airborne contamination removal should be carried out. Backup equipment should be maintained for emergency ventilation requirements for operating rooms [17].

In Europe, various approaches were employed to build HVAC systems in hospitals. The UK’s guidelines are more like the American ones whereas the EU has its own set of regulations based on German, Austrian and Swiss standards. The Robert Koch Institute in Germany produced a new hygiene guideline in 2000 that advocated unidirectional airflow ventilation from a suitable ceiling size. The German Society for Hospital Hygiene developed technical guidance for HVAC systems in hospitals which entailed the elimination of air contamination near the operating table and equipment that could cause direct or indirect surgical site contamination. Vertical low-turbulence displacement flow with an intensity of less than 5% is regarded as suitable for operation. Laminar vertical airflow is also advised [18]. Using HEPA filters in operating room ventilation is akin to applying cleanroom technology requirements to hospitals.

Dust-spot testing and monitoring of the pressure differential across the filters can achieve an improvement in air quality in operating rooms. Few countries have specified bacterial thresholds in traditionally ventilated operating rooms, but most advise 20 air changes per hour to achieve a set number of colony-forming units (cfu). In the UK, an empty operating theater should not exceed 35 cfu/m^3^, and during operations should not exceed 180 cfu/m^3^. Within 30 cm of the wound, the limit is established at 10 cfu/m^3^. These parameters are not always simple to accomplish during surgery because numerous factors affect the microbial load in the air. Air sampling in operating rooms is not standardized in either method or frequency. Volumetric air sampling uses a range of devices, making the correlation of results problematic [19].

Numerous analytical techniques for detecting particulates and identifying bioaerosols have been developed to provide an effective and reliable assessment of air quality. While commercial particulate matter analyzers for larger particles (PM2.5 and PM10) are widely available, fine particles (0.1–0.7 m) and ultra-fine particles (0.055–0.1 m) are harder to detect. However, specialized types of air sensors can be used to keep a continuous check on the operating room environment [20]. Bioscience-viable surface air samplers can also be used to detect microbial load [21].

The extensive use of air filters in closed spaces during the COVID-19 pandemic urges consideration of the environmental burden associated with these means in operating settings. While their use has a major potential to mitigate the spread of viruses, environmental cost-benefit assessments should support any decision to use them in the long term.

### 3.3. Application of Previous Recommendations during COVID-19 OR and What Are the New Steps Forward

As stated earlier in this review, the elaborate guidelines by the CDC addressing surgical cases with tuberculosis can be upheld in the setting of COVID-19, with the usage of N95 respirators, clearing the air of microbial load after the completion of surgery on an infected patient, placement of a filter between the anesthesia circuit and the patient’s airway, implementing use of HEPA filters and allowing enough time to elapse after the surgery for clearance of the environment, and scheduling them as the last cases of the day [16].

According to the guidelines by The International Society for Infectious Diseases, updated in February 2018, surgical clothing, head covering, and masks are necessary in the central area of an operating room, where exposed sterile equipment or scrubbed personnel are present. OR ventilation should filter air at a rate of at least 20 air changes per hour, with at least four of those being fresh air. If resources permit it, this air should be filtered with a high-efficiency filter (HEPA). The operating theater should be cleaned and disinfected according to a strict schedule; for example, flooring should be washed once a day and at the end of each operation. Between procedures, surfaces and all surgical items (e.g., tables, buckets) must be cleaned. Spills of blood or bodily fluids should be cleaned up right away. Because roofs and walls are significantly contaminated only in rare cases, it is normal to clean them twice a year. These recommendations are still pertinent and can be duly applied to the COVID-19 OR setting [22]. However, more stringent regulations for masking, cleaning the room and air filtration can be of use.

### 3.4. Operative Treatment for Patients with COVID-19 and Other Airborne Pathogens

The World Health Organization published guidelines on Infection prevention and control during health care for probable or confirmed cases of Middle East respiratory syndrome coronavirus (MERS-CoV) infection, last updated in 2019. The publication states the implementation of spatial separation (social distancing) of at least 1 m between the patient and other personnel, including healthcare workers (when not using personal protective equipment). Moreover, procedures should be performed in an appropriately ventilated room with natural ventilation of at least 160l/s/patient airflow. Alternatively, when using mechanical ventilation, negative pressure rooms with at least 12 air changes per hour and a controlled direction of airflow should be set up. This is different from regular circumstance guidelines, which entail the use of positive pressure rooms, as opposed to negative pressure rooms recommended in the setting of respiratory infections [23].

In updated technical guidelines published by the World Health Organization during the COVID-19 pandemic, a section addressing surgical procedures in suspected or confirmed COVID-19 patients is included. It emphasizes the usage of a negative pressure room for aerosol-generating procedures as well as anesthesia and intubation. Healthcare workers must don a particulate respirator accompanied by protective eyewear, gown and gloves. If a negative pressure environment is not provided, intubation should occur in the operating room where the procedure will be performed. Designated rooms for COVID-19 surgical cases should be identified, limited to those patients, and kept separate and not in proximity to other theaters. Staff must be limited to essential personnel. Standardized operating should already have a sufficient ventilation rate (15–20 air changes per hour) and their doors should always stay closed during procedures [24].

The COVID-19 pandemic has substantially impacted surgical delivery. Elective surgeries have suffered the most, being postponed or canceled. More than 30 million procedures, or 80 percent of elective non-oncological surgeries, had to be postponed in the first 12 weeks of the pandemic [25]. The COVIDSurg Collaborative used a predictive model to estimate the fall in global elective surgeries resulting from the pandemic: during the 12-week peak of disruption caused by COVID-19, the best approximation was that 28,404,603 operations would be rescheduled or canceled. Most procedures would have been intended for benign disease (90.2%). According to the estimations, the cancellation rate during a 12-week period would be 72.3%; 81.7% of procedures for benign conditions, 37.7% of cancer operations, and 25.4% of elective cesarean sections would be canceled or postponed globally. It would take a mean of 45 weeks to handle the volume of backlog procedures caused by COVID-19 disruption if countries increased their regular surgical volume by 20% after the pandemic [26].

However, surgical delivery is still being dispensed. Surgical cases include COVID-19-positive patients and those whose status may be unknown in case of emergency procedures. Therefore, precautions need to be heightened. Untested patients should be considered as probably infected with SARS-CoV-2. Acute infection tests are inconsistent, with sensitivity as low as 60% when nose swabs are used and 31% with pharyngeal swabs. Additionally, some asymptomatic individuals can shed the virus, posing a risk of disease transmission. Due to the variability of testing sensitivity, the high occurrence of the disease, and the likelihood of asymptomatic individuals shedding the virus, every patient entering the operating room should be presumed positive. Therefore, enhanced respiratory precautions and a rigorous check on the operating room environment are imperative during these times. Proximity with the patient and fine aerosol-generating procedures are the most likely situations for SARS-CoV-2 to be acquired by healthcare staff. The virus can also be detected in body fluids; therefore, personnel should exercise precautions to minimize exposure via aerosolization caused by electrocautery and insufflation gas venting during surgery [27].

Pandemic preparedness in the form of structured, effective planning is vital to ensure adequate handling of the challenges that COVID-19 imposes on surgical practice. Prior to the onset of a pandemic, preparation should be made as part of standard hospital planning. A designated surgeon or anesthetist should oversee designing the plan in consultation with infectious disease and control experts and keeping it up to date as new national and international recommendations are published. Timely training of staff should be carried out in the event of the recognition of a possible pandemic threat. Communication between hospital management, other departments and surgical services should be streamlined and made efficient [28].

Establishing intra-hospital paths for ‘clean’ and ‘contaminated’ patient flow is critical to ensure the provision of regular medical services, especially in situations when system-level ‘COVID-19’ and non-COVID-19 hospitals are not practicable, or when the pandemic reaches a point where separate hospital allocation is no longer viable. Before patients can enter the main premises of a hospital, they should be duly screened for symptoms to protect staff. Ideally, all patients undergoing surgical management should undergo COVID-19 testing. In case of emergent procedures, staff should take maximum precautions to avoid infection. Dedicated health resources, personnel and facilities should be specified as early as possible to minimize outbreak progression [29].

SARS-CoV-2 is transmitted through virus-containing droplets or particles (>5 μm, travel < 1 m) and aerosols (smaller particles less than 5 μm, travel more than 1 m), in addition to touching fomites followed by direct contact (touching eyes, nose or mouth). PPE requirements are generally determined by the potential of a procedure to generate aerosols. In areas of elevated risk units such as operating rooms, high-dependency units, ICU, emergency, wards with non-invasive ventilation, rooms with positive pressure ventilation et cetera, it is recommended to wear a respirator (N99 or FFP3 equivalent, valved or un-valved) instead of a surgical mask together with a fluid-repellent gown and a face shield or visor [30].

Surgical plumes from both laparoscopic and open operations have been shown to contain viral and bacterial aerosols. To combat this, the use of a smoke evacuation device may limit aerosol exposure during both open and laparoscopic surgeries. According to an Italian study addressing quality improvement in operating rooms, operating theaters must be equipped with a HEPA filter that operates under negative pressure and with a high air exchange cycle rate. Each healthcare worker should be supplied with the most efficacious personal protective equipment (AAMI-Level-III surgical gowns; double latex-free gloves with an acceptable quality level of <1.0; and FFP3 or powered air-purifying respirator mask with face shield). Rapid sequence intubation should be used during anesthesia. To limit surgical smoke distribution, energy devices should be adjusted to a lower level and used in conjunction with a smoke evacuation switch pen equipped with a disposable HEPA filter. Low pneumoperitoneum pressures and aspiration systems must be supplied during laparoscopy. Aerosols are produced when electrocautery, bone saws, reamers, and drills are utilized in trauma and orthopedic surgical procedures, increasing the likelihood of viral spread. Therefore, they should be used sparingly, and on the lowest possible power setting. The formation of a viral biofilm is one of the potential issues that can arise on such devices. Disposable medical equipment should be used wherever possible. Sharp injuries to PPE should be strictly avoided. Disposal of blood, secretions, pathological specimens, or any bodily secretions should be in sealed double bags. Before being sent to any laboratory, all obtained specimens should be secured in a biohazard bag in the operating room and then in another biohazard bag in the containment room. Proper labeling of the specimens should also be ensured [31,32,33].

Personnel or ward nurses involved in pre-, peri- and postoperative care of COVID-19 patients also require heightened protection. Transfer from the ward to the operating room should be performed while wearing complete PPE (well-fitting N95 mask, goggles, or face shield, a splash-resistant gown, and boot coverings). A transport ventilator should be utilized for patients being transferred from the ICU. Ceasing gas flow and clamping of the endotracheal tube should be carried out to prevent aerosolization. Alternatively, a portable ventilator equipped with a HEPA filter between the endotracheal tube and the circuit, as well as a second HEPA filter between the circuit and the ventilator can also be installed [32,33].

Between procedures, the operating room must be sterilized. However, disinfection staff should enter only when sufficient air changes to eliminate pathogens have occurred. Ideally, no additional surgery should be performed in the same OR that day, and the theater should be sterilized with UV radiation. Operative paraphernalia sent for sterilization must be duly labeled and the sterilization unit’s personnel must be informed of the case’s COVID-19 status and handle the equipment while wearing complete PPE [31,33].

### 3.5. Making Operating Theaters Sustainable during the COVID-19 Pandemic

With ever-evolving genetic makeup and the emergence of new variants at a rapid rate every few months, COVID-19 seems to be here to stay. For such a threat that adapts itself to persist, healthcare teams’ response should also evolve and be multifaceted to adeptly deal with this notorious virus. Therefore, devising up-to-date protocols for the management of surgical COVID-19 cases is paramount [34,35].

Some steps that can be undertaken include preoperative screening to prevent transmission between health personnel and patients (however, this should not be the primary technique for controlling dissemination); avoiding admission of COVID-19-positive patients where possible; and transferring confirmed positive patients to a dedicated setup and geographical segregation of areas for COVID-19 patients who are cared for by separate health teams or creating separate flow systems where division is not possible [36].

Many nations have resorted to new solutions in response to global difficulties with the PPE supply chain. Production of PPE that can be reused to favorably impact the economy and environment (28% reduction in natural energy usage and a considerable 93% reduction in the generation of solid waste); tracking of PPE utilization, encouraging usage of re-sterilized equipment and personal ownership of protective components are all measures that are being worked on. The European Association for Additive Manufacturing has amped up the production of tools to assist hospitals dealing with the pandemic and the Sustainable Hub for Innovation, Execution, Launch, and Distribution in England, and the South Wales Additive and Rapid Manufacturing Consortium in Wales have been established to further tackle these issues. Along with developing new equipment, repurposing of current OR equipment has been attempted, such as the adaption of orthopedic helmet systems used for elective arthroplasties, in which manifolds were 3D printed and hoods were sewed onto the helmet. Innovations to build electronic PPE have also been undertaken in addition to leveraging telemedicine techniques to conduct electronic medical screening tests which can cut down on PPE costs and waste, and aid in preservation [37].

As the world adapts to the pandemic, innovations to invigorate pandemic preparedness are surfacing. Doctors in a Mexican hospital have come up with an aerosol box: a transparent structure with openings for the doctor’s hands to perform intubation/extubation/aerosol-generating procedures on patients. The box covers the patient’s head and serves as a physical barrier between the patient’s droplets and the healthcare worker [38].

In terms of ventilation and air control, all tiers of filters in the air supply and exhaust systems should have pressure difference detection and warning mechanisms installed that alert personnel when any component malfunctions. Negative pressure maintenance is pivotal for COVID-19 setups. Airchecks with particulate matter and air quality sensors can also be installed to mitigate spread [39].

Preoperative screening of patients should be duly carried out for the safety of staff and appropriate planning of surgeries; it does not cause substantial delays and aids teams in managing infectious cases in an informed manner. Surgical staff should also be regularly screened to avoid intra-hospital spread and to prevent the exacerbation of an already precarious situation [40,41].

With the advent of COVID-19 vaccines and their widespread availability, a vaccination requirement or pre-operative check can be advised to reduce risk for all parties involved. A modeling study undertaken by the COVID Surgery Collaborative and Global Surgery Collaborative supports the prioritization of surgical patients in vaccination plans and advocates early vaccination, especially for individuals over the age of 70 who require elective surgery, as well as other high-risk populations. This could aid in ensuring the safe resumption of elective surgery services, especially in areas where immunization of the entire population could take years. Furthermore, immunization is anticipated to reduce postoperative pulmonary complications, lowering overall healthcare expenses and the need for advanced critical care [42].

Specific practices and alternatives to reducing carbon footprint are the following: selecting specific anesthetic gases and minimizing the materials used in surgery can result in the largest carbon footprint savings; maximizing instrument reuse or single-use device reprocessing can reduce the carbon footprint of an average laparoscopic hysterectomy by up to 80%; reducing off-hour energy use in the operating room can also help reduce the carbon footprint per case; and recycling surgical waste can result in less than a 5% reduction in greenhouse gases. Environmentally friendly absorption of anesthetic gases, such as commercial anesthetic gas capture, can also help reduce the carbon footprint [43]. 

## 4. Discussion

The adaptation of surgical care to the COVID-19 clinical workflow has made surgical procedures less climate friendly. As the restrictions on elective surgery are gradually lifted, it is high time surgeons and health authorities reconsidered the integration of climate-friendly practices into surgical routines. A host of international organizations, surgical institutions and research coalitions have created a framework of recommendations or framed research questions that can be further explored in the quest for environmentally sustainable surgery. This is reflected in the literature revisiting major environmental protection treaties that date before the pandemic, such as the Paris Agreement [44,45,46].

Although implementation needs to be country and region-specific, a number of overarching concerns over feasibility need to be taken into account. Surgical services have already been limited globally, notably in low to middle-income countries, and it has been challenging to establish sustainable services in such regions [47]. This burden is not very much different for the high-income countries as well, as a considerable amount of resources is drained to maintain the quality of the surgical structure [48,49,50,51]. This has been challenging for all the countries irrespective of the resources; however, it has been more difficult for the countries with poor supply chain systems, less capital in reserve and especially when healthcare systems had to manage the weight of the ongoing pandemic [52].

Numerous approaches can be devised to ensure the safety of surgical services during the pandemic. One way is to have an integrated hospital system that can work on the triage of surgical cases based on priority and emergence. A nationwide or region-wide software accessing all the hospitals with information regarding the available operating rooms. This integration can help the referral system to prioritize cases, where urgent or emergent cases can be locally treated and elective cases can be referred to the nearest available operating rooms. This approach can (1) divide the burden of surgical cases; (2) allow the staff to ensure the quality of the environment is COVID free; (3) reduce the surgical volume saturation within a single large center; and (4) make effective pre- and post-operative care. This recommendation is in line with the broader Reduce, Reuse, Recycle, Rethink, and Research (5Rs) framework for sustainable surgery [53,54,55,56].

Another approach could be following a strategy to reduce the operative times during the pandemic. Operative time is already known to be a quality metric and is a major factor that can impact the immediate postoperative outcome. Closed monitoring to ensure the speed of surgery will be an excellent way to control the usage of the operative room and close-circuit interaction of the staff. Preoperative planning should have operative time discussion to ensure that the patient and the staff are in a surgical setting for a finite time only and a third-party independent system should look into the standards of care during this time [57]. Similarly, preoperative checklists may be discussed and filled by the involved parties directly before the beginning of the operation to the extent that patient safety is not compromised [58]. Certainly, this recommendation is subject to the prolongation of surgical time for teaching purposes in surgical departments with a teaching function [59,60].

The present review is subject to a number of limitations. The available studies and gray literature presented significant heterogeneity in terms of reported variables and outcomes. Hence, a comparison of absolute values of carbon equivalents or amounts of generated waste was not feasible. Similarly, depending on the country of origin of each study or set of recommendations, it is understandable that different legislative boundaries regarding environmental pollution are set. In the same sense, the availability and environmental classification of equipment used in different countries may differ significantly depending on the country’s financial capacity and commitment to international standards of climate neutrality. Therefore, the authors attempted to detect a common ground of knowledge regarding the carbon footprint of healthcare and relevant recommendations that can be further explored in response to the environmental burden of the adaptation of healthcare to the pandemic.

This review comprehensively evaluated the available literature and reported a wide variation because of various reasons related to region and operative time. This review mentions that the lowest CO_2_ emission was recorded for a cataract procedure in the US, and the highest emission for a robotic hysterectomy in the US. While these numbers may be useful in understanding the heterogeneity in CO_2_ production, it is also noteworthy to know that not all settings are similar and variation will remain because of the reasons mentioned earlier. A meticulous understanding of the CO_2_ emissions based on the machinery used, the country in which the surgery is conducted and the factors that are related to reducing operative times, are all necessary and must be accounted for. Also, despite a wide variation reported, it is necessary that the goal should be net zero CO_2_ emissions, which may be only virtually possible but that should stay as the primary aim

It is noteworthy to mention that despite the current understanding of the growing global carbon footprint and its current production in surgical settings, it is necessary for a variety of steps for which not many radical steps can be taken right away. These steps include many life-saving events or materials that prevent infections, enhance surgical safety and, overall, benefit both the patients and the surgical staff. The necessary waste requires further evaluation and assessment with a focus on finding alternative materials, or further research is required that can devise methods that can ensure there is minimal to no necessary waste. However, unnecessary waste should be identified as early as possible so they can be labeled accordingly, and can be removed from practice. Furthermore, regarding the necessary waste, recycling is an essential step that should be the focus of research and development and all personnel involved in surgical care have a thorough understanding of these waste types.

## 5. Conclusions

The adaptation of surgical care to infection control during the COVID-19 pandemic has considerable ecological implications. This calls for action towards climate-friendly surgical practices. In terms of research outcomes, suggested potential interventions range from using recyclable personal protective equipment to rationalizing the resources used for sanitation and air quality improvement. In infection control settings, refined isolation protocols and surgical planning can help curb the utilization of non-recyclable resources and energy. Paving the way towards climate-friendly surgery is a worthy endeavor with a major potential to improve surgical practice and outcomes in the long term. 

Further research can be conducted to identify specific interventions that can be implemented to achieve climate-friendly surgical practices and to evaluate the impact of these interventions on surgical outcomes and the environment.

## Data Availability

No data were generated.
